# Protecting endangered megafauna through AI analysis of drone images in a low-connectivity setting: a case study from Namibia

**DOI:** 10.7717/peerj.13779

**Published:** 2022-08-03

**Authors:** Alice Hua, Kevin Martin, Yuzeng Shen, Nicole Chen, Catherine Mou, Maximilian Sterk, Berend Reinhard, Friedrich F. Reinhard, Stephen Lee, Sky Alibhai, Zoe C. Jewell

**Affiliations:** 1 School of Information, University of California, Berkeley, Berkeley, California, USA; 2 Department of Conservation Biology, University of Göttingen, Göttingen, Germany; 3 Kuzikus Wildlife Reserve, Windhoek, Omaheke, Namibia; 4 Army Research Office, Durham, North Carolina, USA; 5 Nicholas School of the Environment, Duke University, Durham, North Carolina, USA; 6 WildTrack Inc., Durham, North Carolina, USA

**Keywords:** Rhino monitoring, AI, Drones, Namibia, Remote sensing, Non-invasive, Remote area monitoring, YOLO, IoT devices, UAVs

## Abstract

Assessing the numbers and distribution of at-risk megafauna such as the black rhino (*Diceros bicornis*) is key to effective conservation, yet such data are difficult to obtain. Many current monitoring technologies are invasive to the target animals and expensive. Satellite monitoring is emerging as a potential tool for very large animals (*e.g*., elephant) but detecting smaller species requires higher resolution imaging. Drones can deliver the required resolution and speed of monitoring, but challenges remain in delivering automated monitoring systems where internet connectivity is unreliable or absent. This study describes a model built to run on a drone to identify *in situ* images of megafauna. Compared with previously reported studies, this automated detection framework has a lower hardware cost and can function with a reduced internet bandwidth requirement for local network communication. It proposes the use of a Jetson Xavier NX, onboard a Parrot Anafi drone, connected to the internet throughout the flight to deliver a lightweight web-based notification system upon detection of the target species. The GPS location with the detected target species images is sent using MQ Telemetry Transport (MQTT), a lightweight messaging protocol using a publisher/subscriber architecture for IoT devices. It provides reliable message delivery when internet connection is sporadic. We used a YOLOv5l6 object detection architecture trained to identify a bounding box for one of five objects of interest in a frame of video. At an intersection over union (IoU) threshold of 0.5, our model achieved an average precision (AP) of 0.81 for black rhino (our primary target) and 0.83 for giraffe (*Giraffa giraffa)*. The model was less successful at identifying the other smaller objects which were not our primary targets: 0.34, 0.25, and 0.42 for ostrich (*Struthio camelus australis*), springbok (*Antidorcas marsupialis*) and human respectively. We used several techniques to optimize performance and overcome the inherent challenge of small objects (animals) in the data. Although our primary focus for the development of the model was rhino, we included other species classes to emulate field conditions where many animal species are encountered, and thus reduce the false positive occurrence rate for rhino detections. To constrain model overfitting, we trained the model on a dataset with varied terrain, angle and lighting conditions and used data augmentation techniques (*i.e*., GANs). We used image tiling and a relatively larger (*i.e*., higher resolution) image input size to compensate for the difficulty faced in detecting small objects when using YOLO. In this study, we demonstrated the potential of a drone-based AI pipeline model to automate the detection of free-ranging megafauna detection in a remote setting and create alerts to a wildlife manager in a relatively poorly connected field environment.

## Introduction

African megafauna are some of the most visible icons of the current decline in biodiversity and as a consequence suffer poaching (illegal killing) for their body products including horn (rhino), tusks (elephant) and flesh (large ungulates). These products have considerable value. For example, rhino horn is used in Asian traditional medicine, and was reported as selling for around $65,000 per kg (Witter & Satterfield, 2019). To disrupt poaching, wildlife managers must deploy effective protection measures. This in turn depends on the availability of reliable data on their numbers and distribution.

Traditional megafauna monitoring technologies are unable to cover large areas effectively. Monitoring by small fixed-wing aircraft is expensive and potentially dangerous in field conditions, being the primary cause of mortality in wildlife biologists (Sasse, 2003). Monitoring using fitted instrumentation (*e.g*., collars, tags) is expensive, has poor longevity and presents risks for the animals (Alibhai, Jewell & Towindo, 2001; Alibhai & Jewell, 2001a; Alibhai & Jewell, 2001b; Alibhai & Jewell, 2002). Traditional monitoring approaches using well-equipped game scouts to patrol and observe individual rhinos regularly can work very effectively when funding, expertise and logistics permit. Ground-based camera-traps can also be effective at identifying animals that move past them but are frequently too expensive to be deployed in sufficient numbers for a comprehensive survey. Moreover, camera-traps require complex sampling protocols to be effective and provide poor discrimination where individuals lack discriminating morphological features (Agha et al., 2018). Other ground survey techniques such as the use of footprint identification (Jewell et al., 2020) can provide low-cost and accurate survey results, identify individuals and be integrated easily with anti-poaching patrols. However, they can also be time-consuming. Thus, there is a need for low-cost, automated, landscape-scale monitoring for megafauna protection.

Remote sensing techniques present one possible solution to this monitoring challenge. Commercial satellites now have the resolution to identify African elephants in open habitat at the landscape scale (Duporge et al., 2021) and whales (Guirado et al., 2019), but their resolution is not yet sufficient to accurately count smaller species of megafauna, for example black rhino and large antelope.

In the last two decades, unmanned aerial vehicles (UAVs), or “drones” (also known as remotely piloted aircraft systems, RPAS), have begun to assume an increasingly significant role in wildlife conservation. Wich et al. (2021) provide an excellent general overview of their current use in conservation. Depending on several factors, drones can also be used without disruption to wildlife (Mulero-Pázmány et al., 2017; Borrelle & Fletcher, 2017). They offer both reasonable ground coverage, and sufficient ground imaging resolution to meet the requirements of local conservation managers. Drones also offer an opportunity to collect data inexpensively and are readily accessible off-the-shelf. Some have onboard computers to process data. Borrelle & Fletcher (2017) cataloged 11 studies from 2011 to 2015 that used them to investigate seabird habitats. Wich et al. (2015) demonstrated that drones could be a useful tool in identifying Orang-utan (*Pongo abelii*) nests in Sumatra. These studies have shown that the drones not only provided a cheaper way to gather the data but resulted in less disturbance to the animals than an in-person survey.

Multiple studies have shown drones’ usefulness in protecting rhinos. Mulero-Pázmány et al. (2014) demonstrated the efficacy of drones to inspect fence lines and locate poachers and rhinos on a game park in South Africa. Penny et al. (2019) identified a possible use for as well as a drawback of drones when they successfully used low flying drones to elicit avoidance behavior from rhinos with the goal of keeping them away from highly poached areas (such as near rivers, roads, and park boundaries). Park et al. (2015) developed a machine learning algorithm to develop an optimal route coordinated for drones and anti-poaching units given historical animal movements and poaching activities.

However, the rhino protection studies mentioned assumed that drones will return to base after each flight where the footage will be manually reviewed. The process of curating and compiling data from drone footage can be slow and it requires a trained eye. Mulero-Pázmány et al. (2014) demonstrated an anti-poaching system based on drones taking still photographs. In that study, it took around 45 min for a trained expert to process 500 pictures. If the drone is taking video at two frames per second (as it did in that article), then each hour of processing would only be associated with 5.5 min of drone flight time. Clearly, manual identification has scalability limits.

Other studies have also sought to automate the drone detection of animals. Tuia et al. (2022) give a thorough overview of the state of the art in the area of automated wildlife detection in general. Corcoran et al. (2021) reviewed studies published between 2015 and 2020 that used automated wildlife detection techniques for drone mounted platforms. They determined that the technology had progressed far enough for automated detection to be viable across a wide range of environments and species. They also concluded that convolutional neural networks (CNN) (LeCun, Bengio & Hinton, 2015), when fine-tuned, can be effective in multi-species detection in varied backgrounds. CNN models such as Faster Region-based convolutional neural networks (Faster R-CNN) (Ren et al., 2017) and You Only Look Once (YOLO) (Redmon et al., 2016) are the state-of-the-art for real-time object detection tasks due to greater accuracy and speed over previously mentioned methods (Corcoran et al., 2021).

Running object detection on-board the drone reduces the amount of time needed for a human to review footage, and can also give game managers real-time geo-locations of animals and poachers. Use of UAVs with embedded hardware in pedestrian detection (Zhang et al., 2019) and tracking (Smedt De, Hulens & Goedeme, 2015) paved the way for other related applications such as animal detection. Until recently, animal real-time object detection and alert had not been fully automated on-board drones due to limited processing power, and limited internet connection in the field. Chalmers et al. (2019) showed that streaming video live over the network is possible on some drones using Real-time Messaging Protocol (RTMP). However, in many remote areas there is insufficient cellular network connectivity to support this protocol. Lightweight messaging architecture, such as MQTT, could address this problem by delivering only images of detected animals instead of real time streaming. We offer a provisional, lightweight object detection model as a demonstration of one approach that could satisfy the demand for low-cost drones capable of operating with poor internet connectivity for real-time detection of wildlife. Such drones would carry on-board hardware capable of inferring high-resolution RGB imagery and providing real-time notifications to users.

## Methods

Terminology: refer to Appendix.

### Data collection

**Figure 1:**
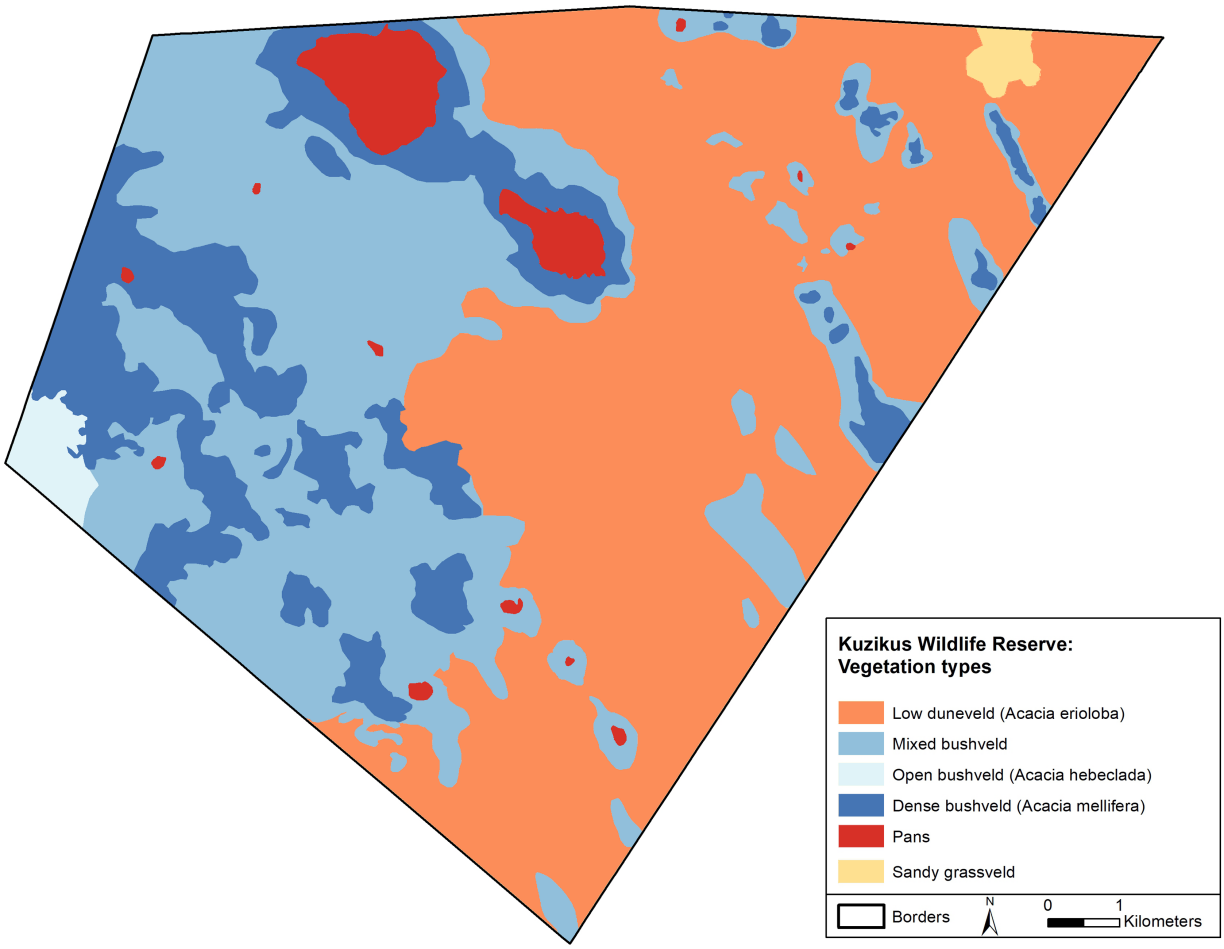
A map of the Kuzikus Wildlife Reserve, Namibia. The Kuzikus Wildlife Reserve is approximately 100 square km. The east side is predominantly Camel thorn (*Acacia erioloba*) and the west is predominantly mixed woodland (*Acacia hebeclada and Acacia mellifera*).

This work was undertaken with permission from the National Commission on Research, Science and Technology of Namibia. The majority of our data were collected from drones flown at Namibia’s Kuzikus Wildlife Reserve. Kuzikus is an area of approximately 100 square km of predominantly mixed Acacia scrub and woodland on the western edge of the Kalahari in Central Namibia (Fig. 1). As one of Namibia’s Black Rhino Custodianship Programme properties, Kuzikus is home to black rhino, giraffe, eland and many other threatened megafauna, affording the opportunity to collect the necessary datasets. Although our primary megafauna focus in this study was rhino, we also collected images of humans, giraffe, ostrich and springbok to provide species breadth for classification and to help reduce rhino false positive detection rate.

We gathered images and 4k videos of rhino and other megafauna from two different drones piloted by Kuzikus personnel. Consistent with Kuzikus policy, drones were flown at an altitude which did not disturb the animals. Avoiding disturbance is dependent on many factors including the sensitivity of the target species to aerial noise, wind speed and direction, model of UAV and flight characteristics (Mulero-Pázmány et al., 2017; Tuia et al., 2022). To collect still images we used a fixed-wing SenseFly eBeeX aircraft, equipped with an Aeria X camera at 24 MP resolution, flown 70 m above ground level (AGL). For video collection, we used a Skydio 2 quadcopter, equipped with a Sony IMX577 camera at 12 MP, flown at 30 m AGL. We extracted still images from 4k video at sufficient resolution for analysis (Table 1). We also used data from Kuzikus which was collected in the framework of the semi-arid savannah habitat mapping project (SAVMAP). The SAVMAP data was collected opportunistically over a 5-year period during a range of seasons from extreme dry to extreme wet conditions, and at different times of the day to include all daylight contrast variations (Reinhard et al., 2015). The Kuzikus training dataset includes images and videos with varying drone altitude AGL, camera capture angles and background conditions. This variance in the training dataset lessens the risk of overfitting.

**Table 1:** Data sources.

Source	Size (px)	Count	Altitude AGL (m)
Drone footage from Kuzikus	4,000 × 3,000	2,131	30–70
	6,000 × 4,000		
	3,840 × 2,160		
	4,608 × 3,456		
Drone footage from Youtube videos	1,280 × 720	667	10–20
	1,415 × 588		
	1,920 × 1,080		
	1,518 × 1,113		
Drone footage from Parrot Anafi	3,840 × 2,160	266	30–70
Still cam footage from San Diego Zoo	1,280 × 720	31	Ground level
Offline & Online augmented	501 × 282	192 & 14,534	Generated from 30–70 m drone images

**Note:**

The source of each data type, the size of images therein, the number of images and the altitude above ground level (AGL) at which they were collected.

**Figure 2:**
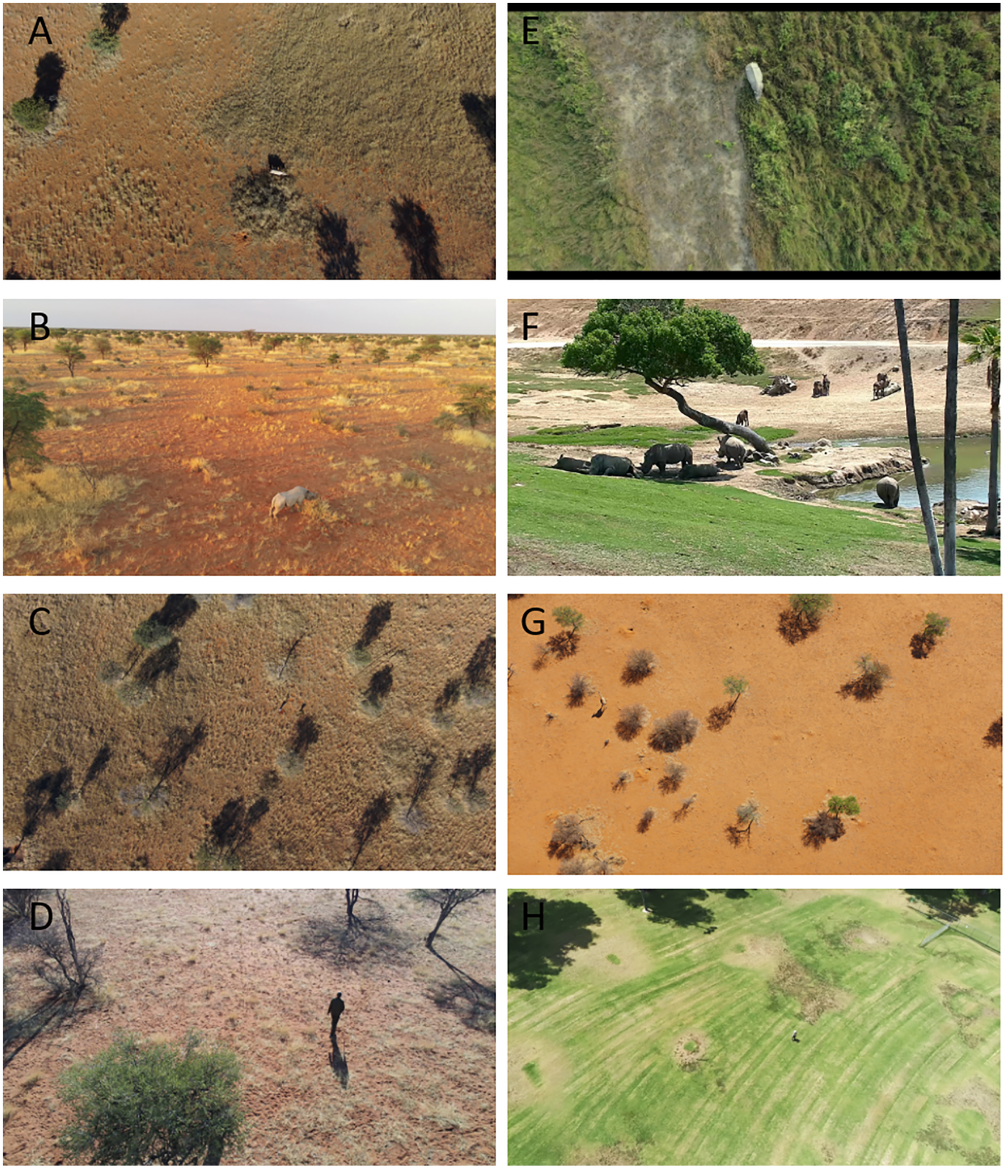
Example drone images from Kuzikus, San Diego Zoo and Almansor Park. (A) Black rhino from Kuzikus captured by senseFly eBeeX drone at 70 m AGL. (B) Black rhino from Kuzikus captured by Skydio 2 quadcopter at 30 m AGL. (C) Giraffe from Kuzikus captured by senseFly eBeeX drone at 70 m AGL. (D) Human from Kuzikus captured by Skydio 2 quadcopter at 30 m AGL. (E) Indian rhino from an open-access Youtube video. (F) Southern White rhino from San Diego Zoo captured by open-access still cam. (G) Giraffe from Kuzikus captured by senseFly eBeeX at 70 m AGL. (H) Human from Almansor Park captured by Parrot Anafi at 30 m AGL. Credits: Kuzikus Wildlife Reserve (A, B, C, D & G); www.YouTube.com, open-source (E). San Diego Zoo (F); Almansor Park, CA (H).

To further augment our dataset, we gathered data from other sources outside of Kuzikus. They provide different angles (top-down or profile view), terrain and background conditions (dry, wet, morning, late afternoon) and altitudes (30 m to 70 m AGL). A richer dataset will result in a more generalizable model that can perform consistently across different terrains, seasons and altitudes. We collected additional rhino drone footage in person as well as from the live still cam at San Diego Safari Park (San Diego Zoo, 2021). We also flew drones at a local park (Almansor Park located in California, USA) to get additional human data. Lastly, we used open-access drone videos of rhinos on YouTube for additional rhino data points. Table 1 details the sources of data used, the image size, the count and altitude AGL at which data was collected. In addition, Fig. 2 shows examples of drone images.

### Data pre-processing

**Figure 3:**
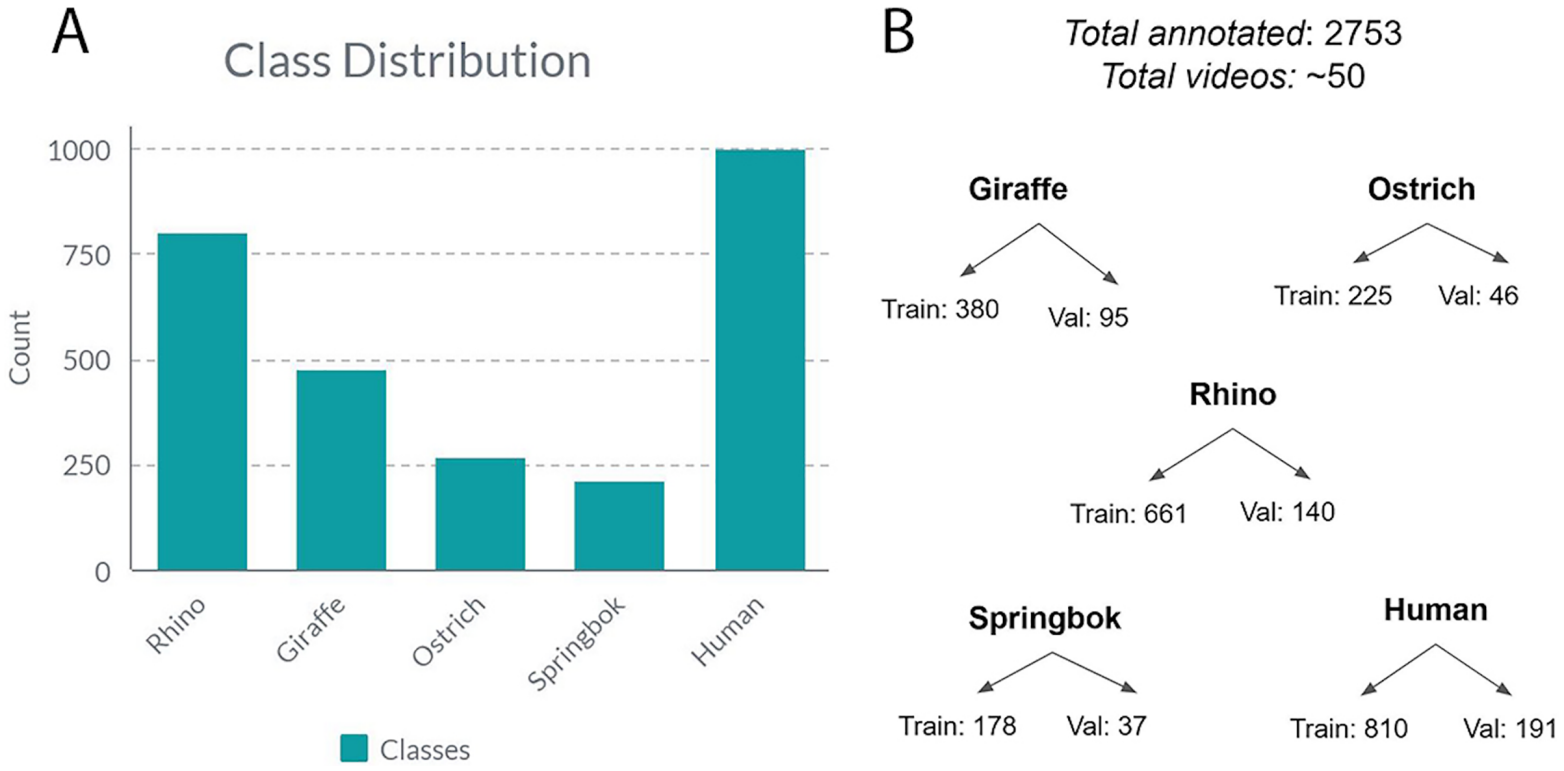
The class distribution for all the annotated frames from all videos. (A) The class distribution for five classes. (B) Class distribution into training and validation sets.

Before we could begin training our model, we needed to break the video in the dataset down into individual frames and then label the images with bounding boxes. First, we used the OpenCV and FFmpeg Python libraries to programmatically extract frames from video. Then we used MakeSense.ai (Skalski, 2019) to manually label bounding boxes for the five species classes, namely: rhino, giraffe, ostrich, springbok and human. We also adopted a semi-manual quality control (QC) process where we used a Python script to automatically process all our images and label files and output a QC image for each input image. We then manually inspected the QC images for bounding box misalignments and relabeled as needed. We aimed for an 80:20 split between training/validation sets. We kept each video either entirely in the train or validation set, but not both to avoid data leakage where the model simply memorized the feature space for the given video and thus predicted well on the validation set because it had seen near identical frames (belong in the same video) in the train set (Brownlee, 2020). Figure 3 shows the class distribution breakdown. We also curated a test video from a holdout set of videos from the above sources. The video is 2:34 min long, contains 145 frames of five different target species captured at flying altitudes ranging from 30–70 m AGL, under various angles, lighting and terrain conditions.

#### Offline augmentation

**Figure 4:**
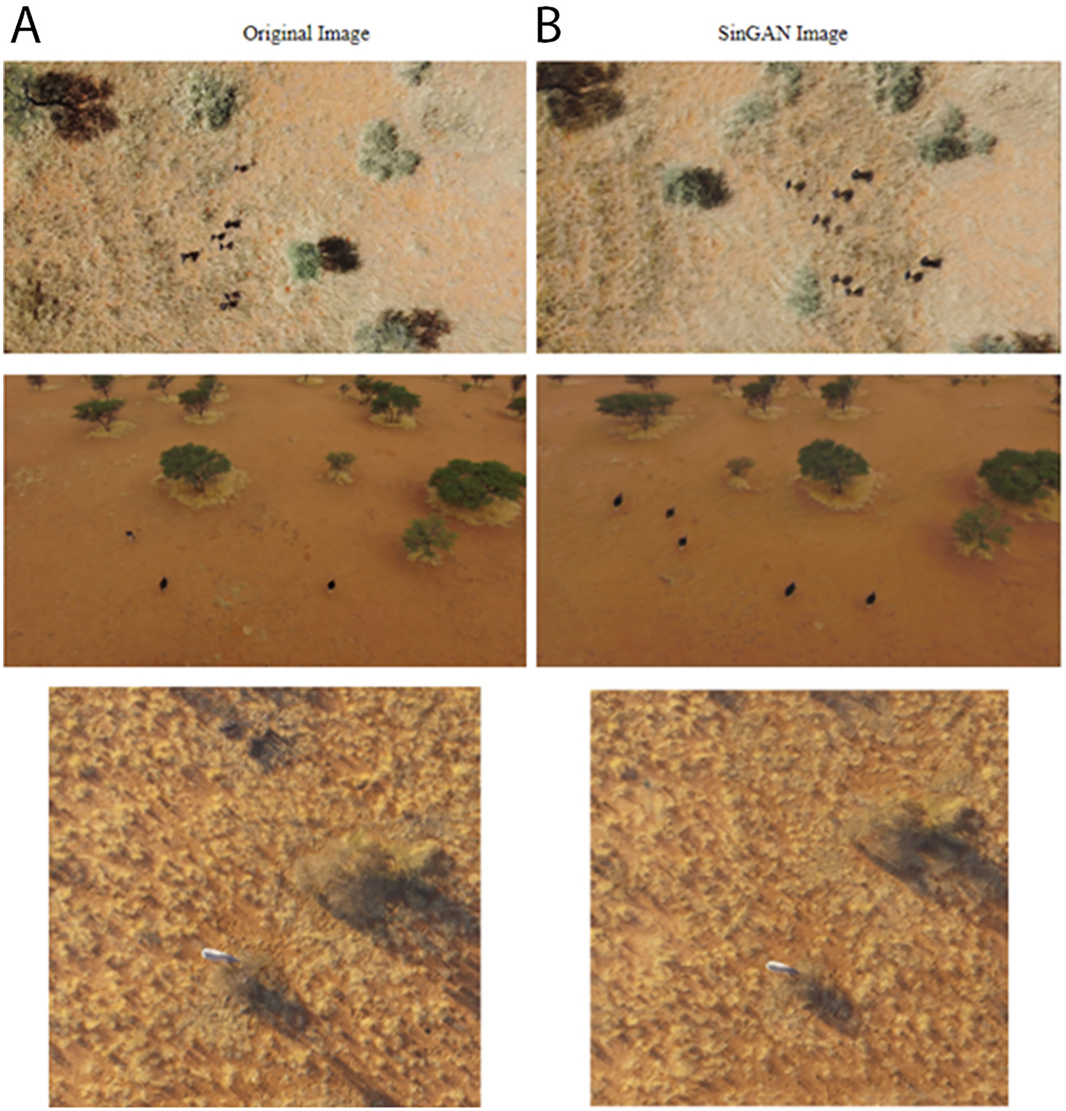
Offline augmentation using SinGAN examples. (A) Original images. (B) SinGAN images of ostrich and rhinos.

**Figure 5:**
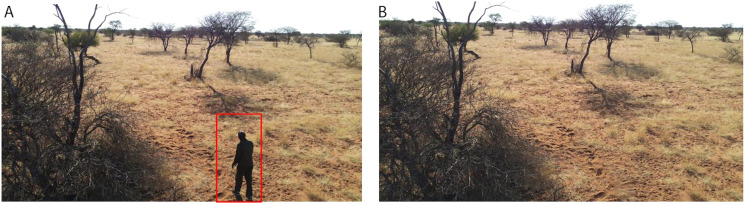
Offline augmentation using photoshop. (A) Original image. (B) Photoshop image where the object of interest is removed. Credit: Kuzikus Wildlife Reserve.

A larger dataset is generally known to increase performance of machine learning models. To increase the size of the training dataset, we created additional synthetic images using two methods, a type of Generative Adversarial Network (GAN) (Goodfellow et al., 2014) and Adobe Photoshop. For the first type of synthetic images, we chose Single Natural Image GAN (SinGAN) which learns on a single training image and generates similar images with different object configurations (Shaham, Dekel & Michaeli, 2019). This type of GAN model does not require many images to train itself. Many other types of GAN training often require a large amount of training images. SinGAN learns the internal statistics of image patches, rather than the whole image as in a conventional GAN. It then generates patches that when combined, produce realistic images while maintaining the patch statistics. We used it to generate new frames on rhinos (82 images) and ostriches (44 images) on similar backgrounds to an original drone image. These new synthetic images were manually labeled and added to the training data. Figure 4 shows SinGAN examples of synthetic images. Our second type of synthetic images are background images. We used Photoshop to remove target objects from images to generate background images. It is recommended to have at least 10% background images for best training results (Jocher, 2021). Figure 5 shows Photoshop examples of object removal for background images.

#### Online data augmentation

**Figure 6:**
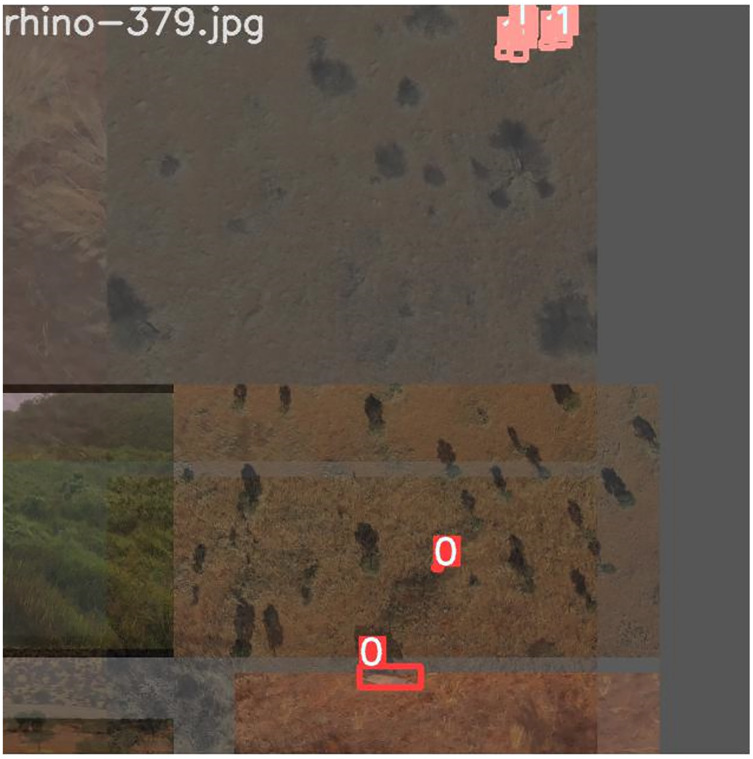
Online augmentations using the Albumentations library. A training image that utilizes the mosaic and flip augmentations. Note that all images are flipped about their X-axis and different base images are arranged next to each other to form the single input image. Label 0: rhino class; 1s: giraffe class; 2s: ostrich class; 3s: springbok class; 4s: human class.

By using online data augmentation, we were able to increase the number and variety of training images that the model was exposed to without having to do additional labeling or manual manipulation. This helped the model generalize better to unseen data and avoid overfitting. To do this, we automatically transformed existing images using the Albumentations library (Buslaev et al., 2020) in the model training pipeline. The specific techniques used included horizontal and vertical reflections of a portion of the images (random flipping), applying a slight blurring effect (blurring), applying a transparency effect to images and layering them (mixup) (Zhang et al., 2017) and using a 2 by 2 array of images for input (mosaic) (Bochkovskiy, Wang & Liao, 2020). Figure 6 shows an example of these online augmentation techniques. We observed an increased model performance on the validation set as a result of these combined augmentations. Furthermore, we implemented a third type of image augmentation called tiling. We describe our implementation of tiling below.

### Tiling

A significant proportion of images in our dataset suffered from a common issue known as the “small object detection problem” where objects of interest were small relative to the size of the image. We addressed this by deploying tiling on the higher resolution training images of our target classes. In our study we did not implement tiling during the inference phase.

Tiling is a process of breaking up a large image into smaller pieces, then running those pieces through the model. It is a widely used technique in computer vision. DuPorge et al. (2021) employed this technique in their study detecting elephants from high resolution satellite images. Bounding boxes in these smaller tiled images are retained and scaled accordingly so that the object of interest remains boxed. Generally, tiles are overlapped on the image so that objects at the border between tiles are kept intact. This solves the small object problem because the smaller tiled images do not need to be compressed to be fed into the model and thus, they maintain their full fidelity. The higher resolution images, when tiled, contain more detailed information and training on them results in a model that makes more accurate predictions (Růžička & Franchetti, 2018).

**Figure 7:**
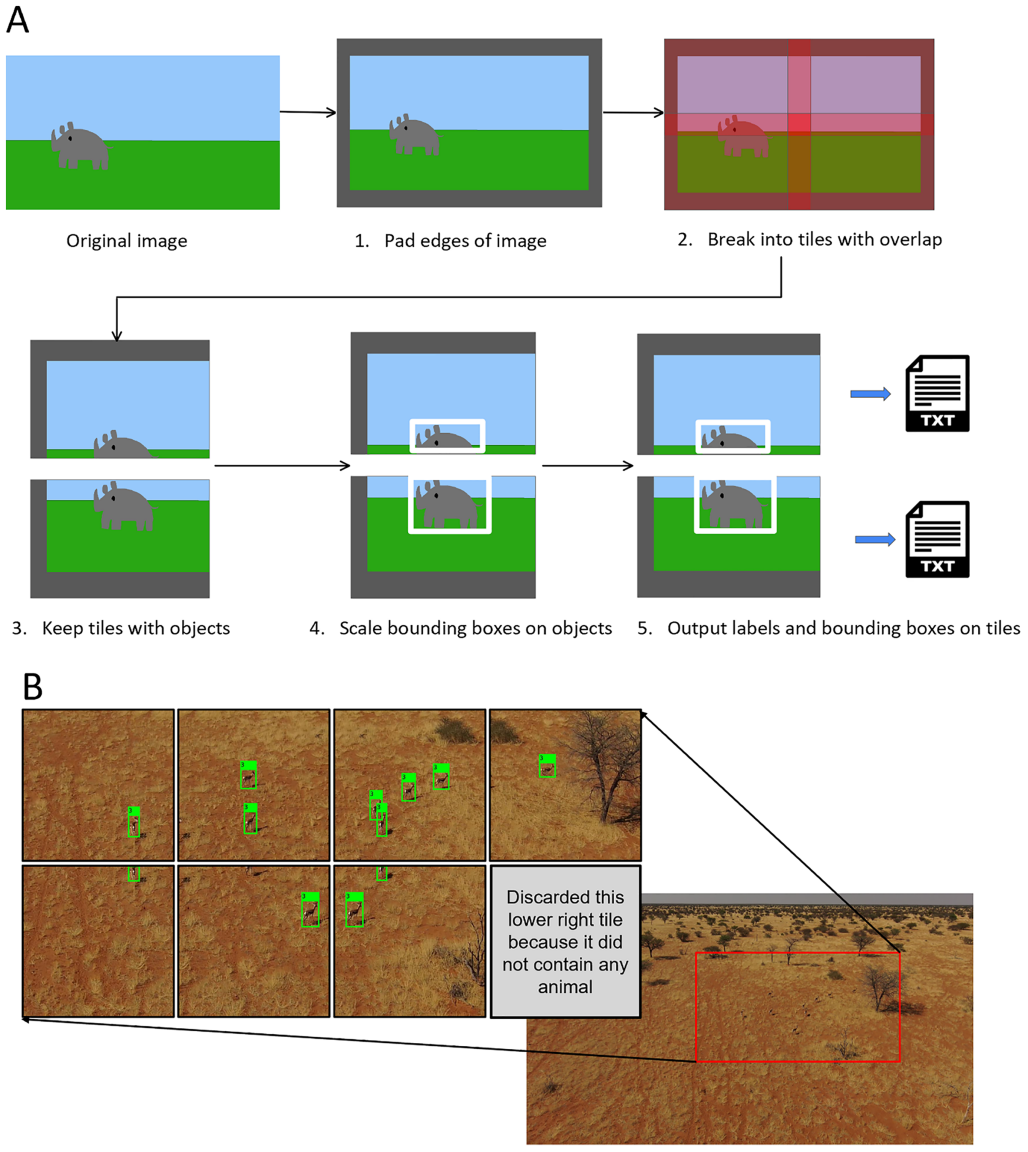
Augmentation using tiling. (A) Tiling process schematic. (B) Example of a tiled springbok image (original image 3,840 × 2,160 px).

In this study we implemented tiling during the training phase and applied it to our high-resolution inputs (any images bigger than 1,280 px). This allowed us to significantly augment the dataset. First, we considered that a high quality 720p video frame comes in at 720 × 1,280 px in size. With such video quality, small objects would still likely be visible and so we set our object detection model to take in images at 1,280 px. We then tiled our images at 1,280 × 1,280 px with a 320 px overlap between adjacent tiles (Fig. 7). We used a one quarter tile overlap to help prevent objects being lost at the boundary between tiles (Ünel, Özkalayci & Çiğla, 2019). Our tiling also facilitated the partitioning of some of the ultra high-definition images in our dataset (some were 4,000 px wide) yet maintained small objects at a reasonable size.

Although our image sizes varied considerably, from 1,280 to 6,000 px width, the bounding boxes tended to be all clustered around the same size. The majority of bounding boxes (~99%) were under 200 px. The uniform size of the bounding boxes gave us confidence that using a uniform pixel size for the tiles was appropriate. The tiles were large enough that all bounding boxes were captured by one of the tiles in overlapping regions. For example, if we had used a tile size smaller than around 200 px then for our larger bounding boxes each tile might capture only a corner of the rhino and potentially none would have a large enough segment of the bounding box for it to be a complete image usable for training.

In cases where objects were partially cut off by the tile, we decided to only keep the frame for training if more than 50% of the bounding box for the target object was contained on our tile. This prevented potentially irrelevant bounding boxes with limited or no info from appearing at the edges of the tiles (Fig. 7). To implement the tiling, we heavily modified a script from GitHub by Neskorozhenyi (2021).

### Object detection model

Convolutional Neural Nets (CNNs) are state of the art machine learning models often used in computer vision (CV). In the context of CV, a CNN model takes images as input, learns their features (shapes, textures, edges, *etc.*) using multiple layers (convolution, pooling, Relu, *etc.*) and classifies the images. Feature learning involves using different filter (kernel) types and performing convolutional computations on the image pixels (Goodfellow, Bengio & Courville, 2016). In general, CNN-based object detection methods can be further divided into two main groups: two-stage and one-stage. Two-stage detectors first use a network that predicts many regions where bounding boxes are likely to be in an image, then it passes those results off to the actual convolutional network that determines if any of the proposed regions are the object of interest. Faster R-CNNs (Ren et al., 2017) are currently one of the most widely used architectures for these two stage detectors. One-stage detectors put the entire image through a single network and predict bounding boxes and object classes at the same time. The YOLO family of models (Redmon et al., 2016; Redmon & Farhadi, 2018; Bochkovskiy, Wang & Liao, 2020; Jocher et al., 2020) are among the most widely used one-stage detectors. There is a tradeoff between using these models. Two-stage detectors tend to be more accurate but slower while one-stage detectors have lower accuracy but faster performance times (Redmon & Farhadi, 2018).

We utilized YOLO as our real-time object detection model of choice because it has the fastest inference speed, which is essential for real-time video inference on a relatively low powered edge device. The original YOLO paper (Redmon et al., 2016) suggests that YOLO is relatively robust to domain shifts. The authors found that it outperformed Faster R-CNN models in terms of both accuracy and speed when both models were trained on photographic data and used to detect objects in paintings and other artwork. They suggest that this robustness is due to the fact that YOLO sees relative location of objects and their shapes rather than just a small region. This is important because our data comes from a variety of sources where domain shift is a potential concern; we want the model to perform well if the target is at an angle or if the background and lighting are different than the training data.

For the specific implementation of the YOLO algorithm, we used YOLOv5 rather than the earlier versions of YOLO. This was due to YOLOv5’s smaller model sizes, shorter training and inference duration, the flexibility to utilize pre-built optimized models based on image size (Jocher et al., 2020). For example, the YOLOv5l6 architecture, which we used, includes more hidden layers than the YOLOv5s baseline model. The YOLOv5l6 architecture also contains an additional output layer which predicts features at a different scale than the baseline YOLOv5s. These combined features make it work well when ingesting larger images. This implementation also makes it very easy to build even lighter weight models which use half-precision (16-bit floating point or half-precision weights) (Solawetz, 2020). Half-precision was one of the factors that allowed us to produce speedier inference by using less memory for computation. This allows our model to be lighter, and more portable on the edge device where computation resources are limited. We posit that YOLOv5 used in combination with a lightweight messaging component is suitable for our use case of limited network connectivity on the field.

The YOLOv5 model architecture consists of the backbone, neck and head. The backbone is the part of the network used to extract important features such as edges from the given input image. It uses a cross stage partial network (Wang et al., 2020) for this part of the model. The basic extracted features are then sent to the model neck, which is used to generate more elaborate features. YOLOv5 uses a path aggregation network (PANet) (Liu et al., 2018) for the model neck. This architecture is based on feature pyramid networks (Lin et al., 2017a), which help the model to generalize and identify the same object at different sizes and scales. The model head is mainly used to perform the final detection by applying anchor boxes on features and generating final output vectors with class probabilities, objectness scores, and bounding boxes (Rajput, 2020). There is no YOLOv5 paper to date, but we refer to this detailed series of diagrams of the YOLOv5 architecture, which interested parties can inspect (Laughing-q, 2021). We use the default settings for YOLOv5, with stochastic gradient descent (SGD) (Kiefer & Wolfowitz, 1952) as the optimizer, a predefined set of activation functions and BCEWithLogitsLoss from the Pytorch library (Paszke et al., 2019) for the loss function.

We performed model training on EC2 instances from Amazon Web Services (Amazon Web Services, 2021). We utilized different levels of instances (G and P instances, with VCPU utilization ranging from 16 to 96) during the course of model development due to progressive increases in model complexity and dataset size. We iterated over 8 models during our model development process. Our final model was a YOLOv5l6 trained from scratch (without pre-trained weights) for 200 epochs, with a batch size of 32, image size of 1,280. It was trained on a 96 VCPU 8-GPU P-instance from AWS. The model’s best set of weights was used for inference on the hold out test set. Inference was performed on the Jetson NX edge device.

It is important to note that while the model training process was compute-intensive, the model inference process was lightweight and was able to be run on our small Jetson NX edge device. Model training takes many orders of magnitude more compute power than inference. Training requires running the model many times over the training *corpus* while applying on-line image transformations to incoming images and back propagating the results of each run up the model. This process applies the losses of each run to calculate new model weights and improve performance. The output of training is a set of the best model weights. These weights only need to be produced one time from the model training process and are light enough to be run on a much smaller device to produce predictions at inference time.

### Evaluation metric

Our primary evaluation metrics for the model were the average precision (AP) by class and the mean average precision (mAP) for the model overall. These are the most used metrics used to assess object detection model performance. The ability to objectively compare our model results to previous studies allowed us to iterate and benchmark our model development process accordingly. We were particularly interested in increasing the AP of the rhino class as our main object of interest. The performance of rhino and human classes were prioritized over other classes during our model development. This was due to the assumption that our model would be used in the anti-poaching context.

**Figure 8:**
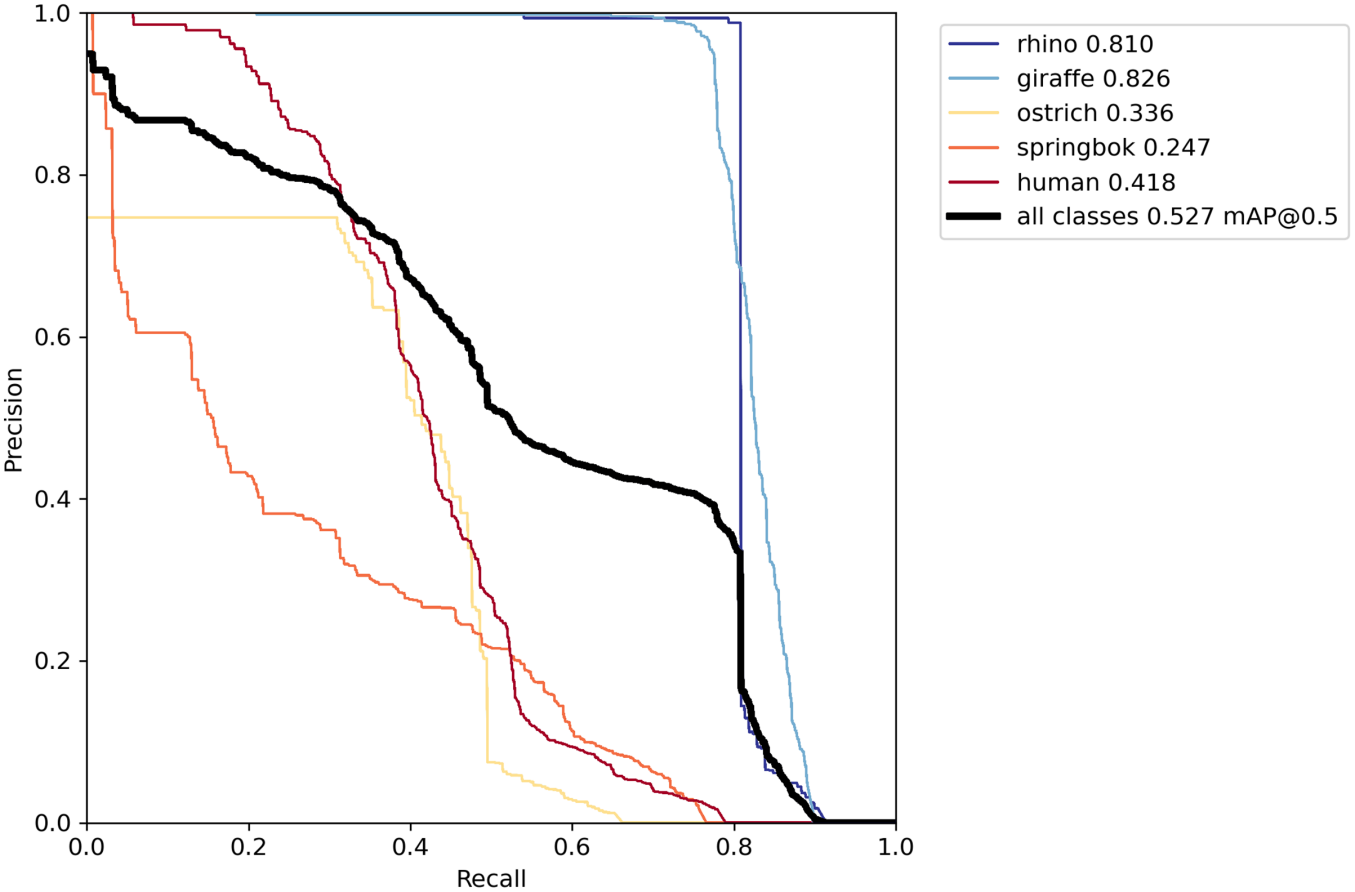
Precision-recall curve on the validation set. The best model using both offline and online techniques yielded a recall of at least 80% for rhino and giraffe classes where their precision rates were highest. Other classes did not perform as well, particularly springbok.

Deriving the AP per class required defining our intersection over union (IoU) threshold. The IoU computes the area of intersection divided by the area of the union between the true bounding box and the predicted bounding box. An IoU of one indicates that the bounding boxes overlap perfectly. We used the IoU threshold of 0.50 (the default threshold for YOLOv5) when reporting our model. In this case detections where positive results are above the IoU threshold are classified as true positives, and those below the threshold are false positives (assuming that the class label is correct). Based on the true positives (TP) and false positives (FP) and false negatives (FN), we derived the precision and recall for all objects to construct the precision recall curve (Fig. 8).

### Model development

For our initial modeling, we used three classes, rhino, human and other animals. Performance was increased when we added additional classes (rhino, giraffe, ostrich, springbok, human) in the next iteration. Our baseline model used the lightest YOLOv5s architecture with an input image size of 992 × 992 px. We did not use offline data augmentation in the baseline model. Final model improvements involved adding synthetic images from SinGAN and Photoshop to the dataset to increase the examples for the rhino, ostrich and background images. We also used additional forms of data augmentation and regularization parameters described above such as mixup, mosaic augmentation and label smoothing (Szegedy et al., 2016). We then tiled images larger than 1,280 px in size and added the tiles to the training dataset. Lastly, the final model used the larger YOLOv5l6 architecture, which ingests images at 1,280 px. The larger input size in our final model allowed us to optimize for small object detection by minimizing the loss in feature space when an ingested large image (*e.g*., 6,000 × 4,000 px) was scaled down by the model.

### Edge implementation

Here we describe a proof-of-concept implementation, where a Jetson Xavier NX is onboard a Parrot Anafi drone and connected to the internet throughout the flight (noted in Discussion). In our study, we did not have the powered edge device attached to the drone itself and running in the field due to budget constraints. We did, however, run model inference on the hold-out test video on our edge device. The device can be mounted on the drone and attached to a power source (Zhang et al., 2019).

In a full implementation where the edge device is mounted on the drone, our pipeline processes the live stream coming from the drone’s camera. In our implementation, the Jetson NX breaks the video into frames, then carries out object detection using the weights and hyperparameters of our best model. We used half-precision weights to speed up the inference time as the model predicts bounding boxes for detected object classes. For each positive detection in each frame, the Jetson NX gathers the drone’s latitude and longitude for the predicted class, the frame with the predicted bounding box(es) and the confidence score. We bundle these metadata for every valid bounding box within each frame. We send them together to avoid mismatching the predicted class and the frame or location that it corresponds to. Only frames with over 50% confidence score are sent to the cloud hosted web app. Note that we generated random coordinates in our data due to rhino safety and privacy concerns, but in reality this would come from the drone’s onboard GPS unit.

The message communication is facilitated by MQTT, a lightweight messaging protocol using a publisher/subscriber architecture for IoT devices. We sent our messages with a quality-of-service score of 1 to ensure the message is delivered at least once (MQTT, 2021). The publisher lives on the Jetson, where it publishes the message bundles generated by our inference process. The subscriber lives on the cloud and listens to these bundled messages, decodes them, and updates our web app. Our web app is hosted by Flask, which lives on a cloud server.

**Figure 9:**
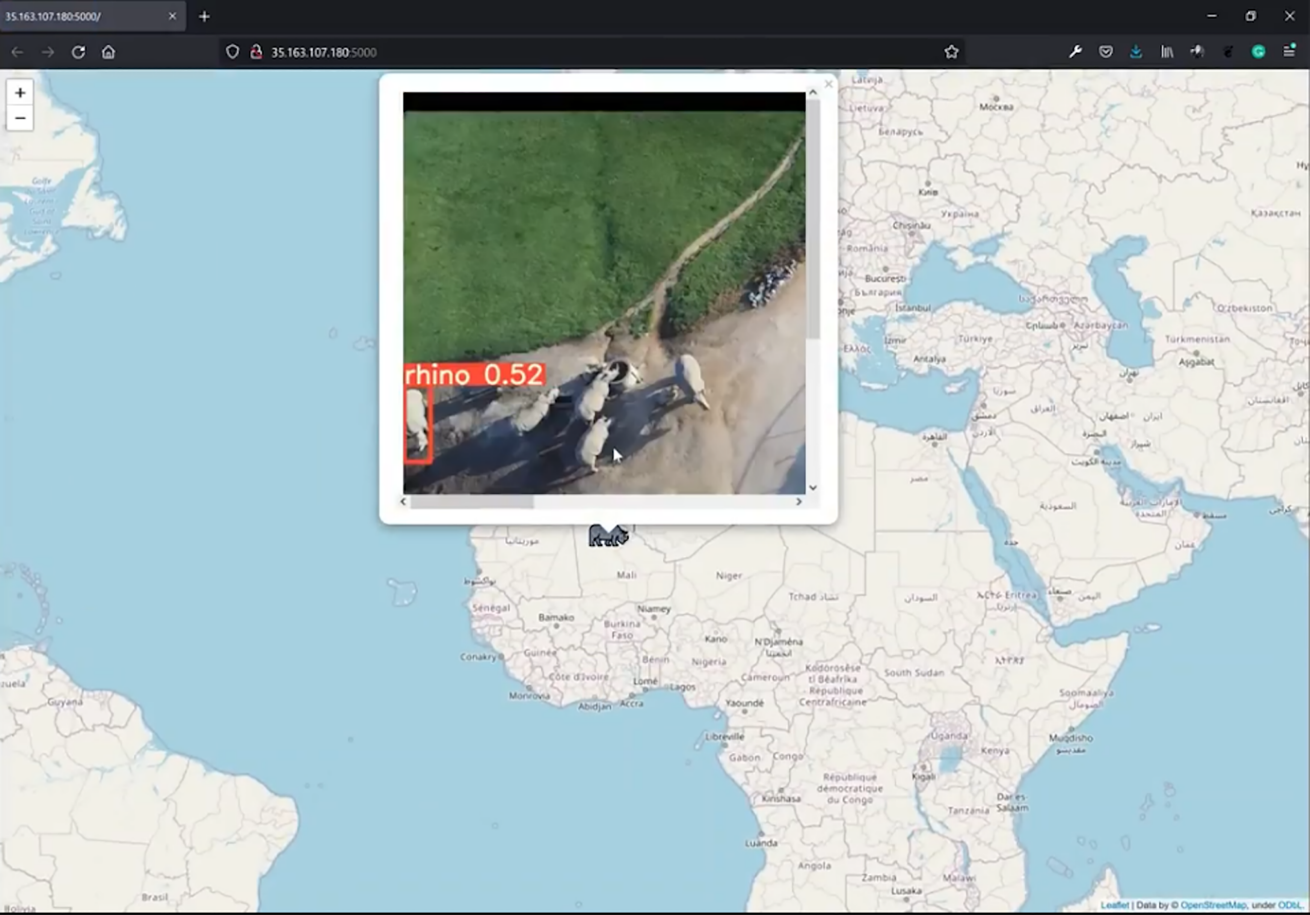
Edge notification system. Sent image of low altitude (30 to 40 m AGL) rhino class. This figure illustrates an arbitrary location. Credit: OpenStreetMaps.

The map within the web app is rendered using Folium, a library used for visualizing geospatial data (Story, 2013). It displays the corresponding icon of the predicted class (rhino, ostrich, giraffe, springbok, human) on the broadcasted location. The user can then click on the icon to see a popup, which contains the frame with the predicted bounding box around the identified object together with its confidence score. Each time the user refreshes the page, the map is updated with the latest result pushed from the Jetson during inference (Fig. 9).

### Project pipeline

**Figure 10:**
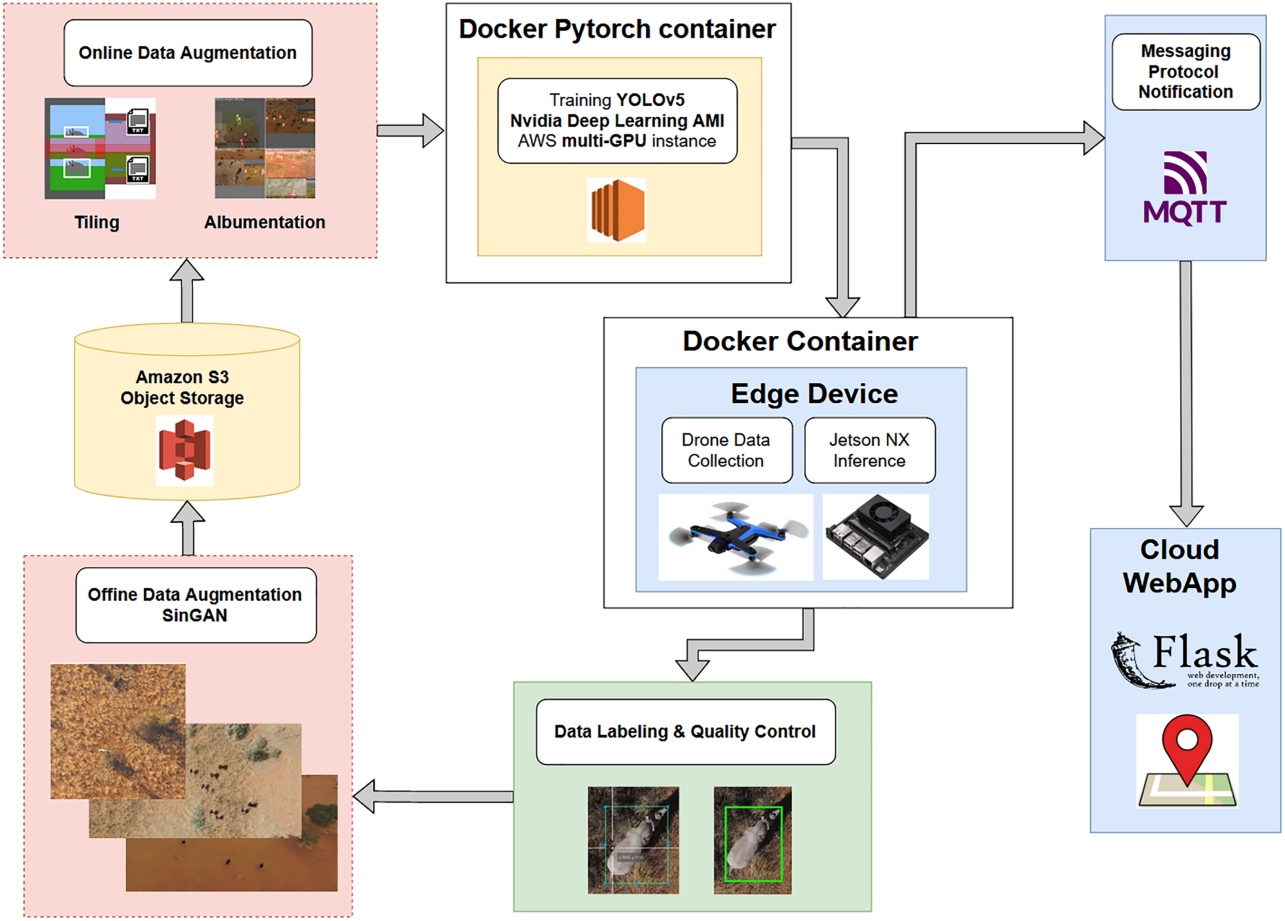
A pipeline schematic from data collection to edge deployment. Data collected by a drone are labeled, subjected to offline data augmentation, stored in the cloud, subjected to online data augmentation using tiling or Albumentations, and trained by YOLOv5 on the drone. The inference is passed through a messaging protocol to a cloud web app and then to a user on the ground.

Figure 10 shows the pipeline schematic from data collection to edge deployment.

## Results

**Figure 11:**
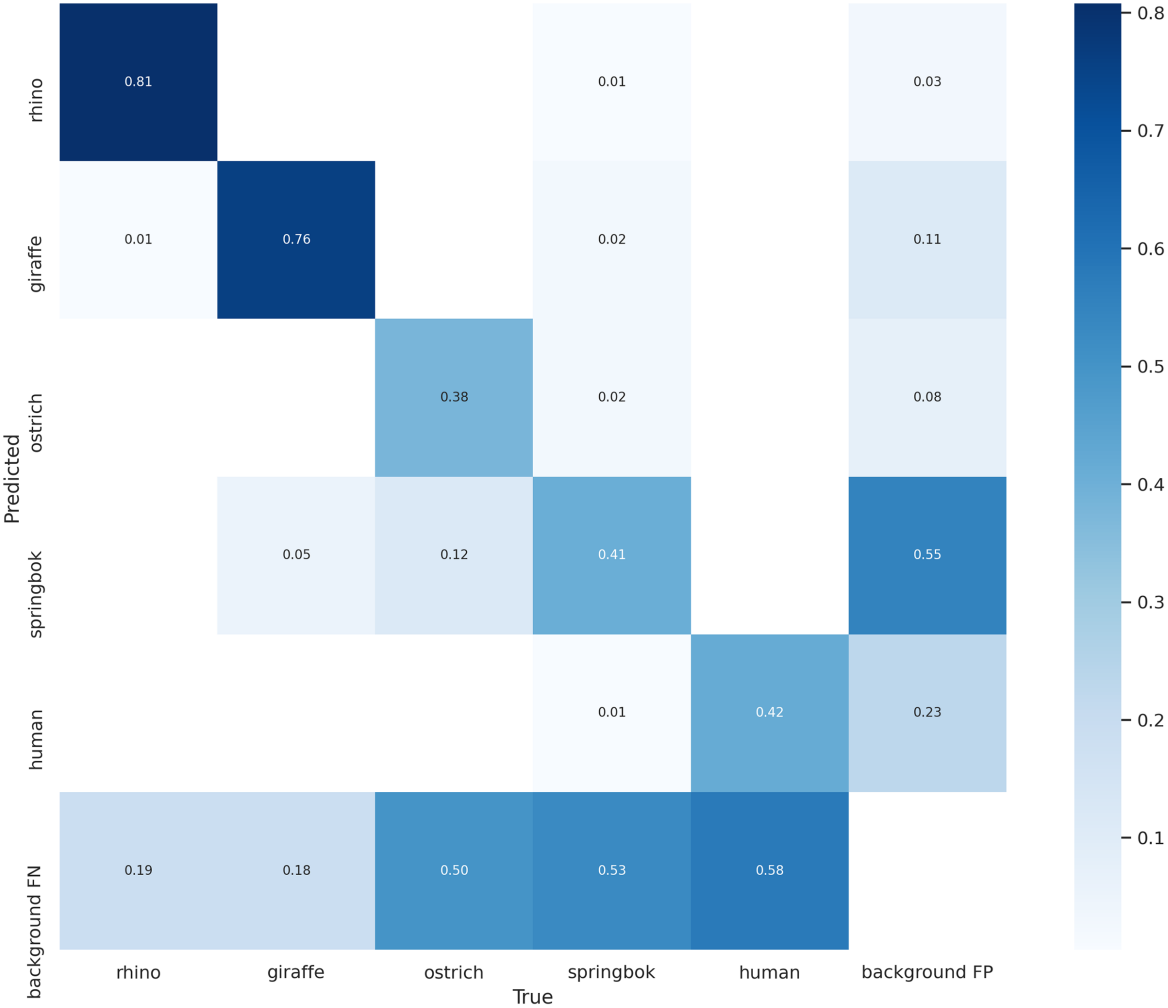
Confusion matrix for the five classes on the validation set. The main target class, rhino, can be predicted accurately 81% of the time. Giraffe class has a similar accuracy. Smaller-sized classes such as ostrich, springbok and human are misidentified as background 50% of the time on this validation set.

Our final model achieved an AP of 0.81 for rhino, 0.83 on giraffe, 0.34 on ostrich, 0.25 on springbok and 0.42 on human for an overall model mAP of 0.53 (Table 2; Fig. 8) on the original un-tiled validation dataset. The overall model mAP score was pulled down by having lower AP scores for the smaller target species. We included the smaller targets because they improved the performance of the main target classes (rhino and human). Figure 8 shows the precision-recall curve and Fig. 11 shows the confusion matrix for our final model. The rhino and giraffe classes were predicted more accurately than other classes. Springbok, ostrich and human classes were often predicted as background.

**Table 2:** Data training results.

Model name	Class	Model	GAN	Online Augs	Img. size	Tiles	Data set size	
3-Class Baseline	Rhino (R) Other (O) Human (H)	YOLOv5s	None	hsv_s 0.7 mixup 0.0 iou_t 0.2 lab_smooth NA	992	None	2,276	62.7 (R) 36.8 (O) 23.2 (H)
5-Class Baseline	Rhino (R) Giraffe (G) Ostrich (O) Springbok (S) Human (H)	YOLOv5s	None		992	None	2,276	67.7 (R) 66.7 (G) 35.8 (O) 7.34 (S) 20.2 (H)
Final Model		YOLOv5l6	Yes	hsv_s 0.0 mixup 0.5 lab_smooth 0.1 A.HorizontalFlip A.RandomRotate90 A.VerticalFlip A.MotionBlur A.MedianBlur A.GaussNoise	1,280	Tiles: 4,390 Tile size: 1,280	7,409	81.0 (R) 82.6 (G) 33.6 (O) 24.6 (S) 41.8 (H)

**Note:**

Model name (three or five classes), class names (species), the YOLO model that trained them, whether GAN was used, online augmentations used, image sizes, tiling, dataset size, the accuracy achieved using average precision metrics AP at IoU of 0.5. Our base model is three-class (rhino, human, other), our second and final models are five-class (rhino, giraffe, ostrich, springbok and human).

Due to a limited dataset, we combined both high and low altitude images in our training set. We saw that inferring on higher altitude images (70 m AGL) resulted in lower mAP scores, and tiling improved the performance significantly as explained in the methods and discussion sections. The human and springbok classes were most challenging to detect (lowest APs) because of the combined altitudes (high and low) in the validation data; they were also smaller in size compared to rhino, giraffe and ostrich and we had a limited number of images for each type of altitude. It is important to note that the image split between the train and validation sets for each class is uneven with regard to the number of high *vs* low altitude images per class. This is because we did not want individual videos to split up in between train, validation and test (see ‘Data pre-processing’ section). In total, we only had 271 images of ostrich and 215 images of springbok as compared to 801 images of rhino and 475 images of giraffe. Table 3 shows the total number of images per class.

**Table 3:** The number of images per species and their corresponding AP@0.5 in the validation set.

Species	Frame count	AP@0.5
Rhino	801	81
Giraffe	475	82.6
Ostrich	271	33.6
Springbok	215	24.6
Human	1,001	41.8

**Note:**

The frame count provides the number of still images extracted from the videos for each species. The AP@0.5 is the average precision for each species at an IoU threshold of 0.5.

**Figure 12:**
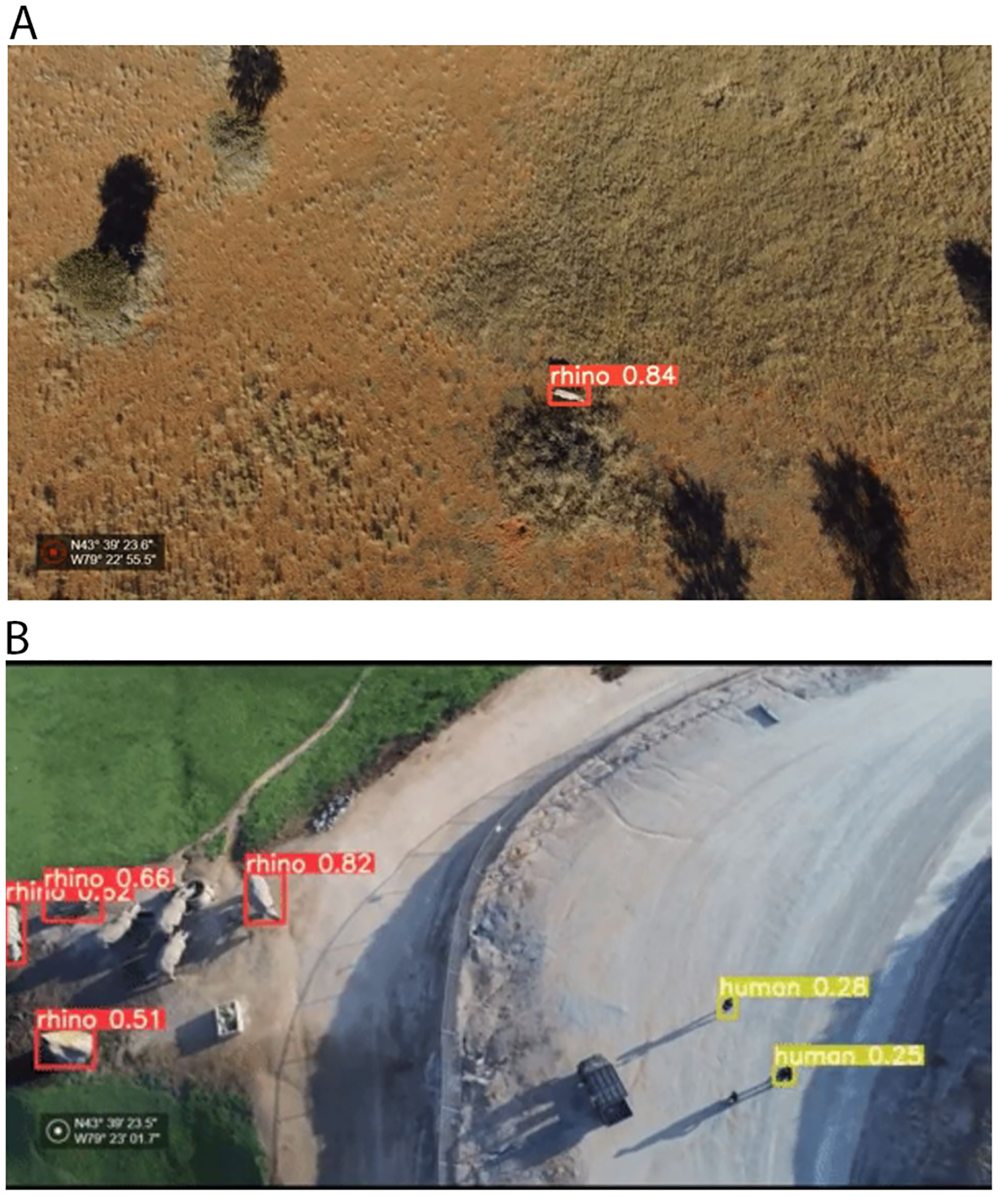
Test set inference result examples. (A) Black rhino from Kuzikus Wildlife Reserve collected by a fixed-wing drone at 70 m AGL on a dry substrate (B) Southern white rhino and humans from the San Diego Safari Park collected by a quadcopter at 40 m AGL on a moist substrate. Credit: Kuzikus Wildlife Reserve (A); San Diego Safari Park (B).

Although we increased the human class images significantly with the additional drone footage at the Almansor Park (for a total of 1,001 images), its highest AP was still below 0.50. We observed that the additional data source provides images of a wet green environment, which is very different from human images at Kuzikus (Fig. 2). By keeping the videos separate in either split, our validation set for the human class ended up with more high altitude and dry background images. Figure 12 shows inferred test images at about 70 m AGL and 40 m AGL, across two different terrain conditions. We had more success with the rhino class potentially due to more even splits of image types and the fact that rhinos are much bigger than humans.

Overall, our results indicated that larger models with larger image size input took significantly longer to train but yielded higher accuracy. They were still able to be run on the edge device. Model performance was increased by utilizing online augmentation techniques such as mixup, *etc,*. and offline augmentation techniques such as tiling, GAN and photoshop. Incorporating specific species, as opposed to “other animals”, improved model performance. Our last model used tiling; this doubled the training dataset size and by far, provided the most noticeable performance boost across four out of the five classes.

## Discussion

### How our results compared with other similar studies

When we consider our results in comparison to other similar studies of drone-based animal object detection models by Chalmers et al. (2019) and Eikelboom et al. (2019), we see that our model performance in terms of AP for objects of interest is comparable to their results. However, our model’s inference speed is orders of magnitude faster and it is able to be run on a lightweight edge device, whereas the other two comparable studies ran their models on more expensive hardware.

The study by Chalmers et al. (2019) is the most comparable of the two. Their model achieved an overall mAP of 0.83 at an IoU of 0.5. They used two classes, rhinos and cars. Given that our focus species was rhino, our most comparable result would be the rhino AP of 0.81. Their target classes were of similar size to the rhino and the giraffe in our model but larger than the ostrich, springbok and human. Thus, they might not have suffered from the same small object problem that we encountered. Furthermore, Chalmer’s model evaluation was done on one type of terrain (in RGB and in thermal images), not varied terrain like in our study. If we were to reduce the number of classes and keep the dataset to one specific terrain, then we would expect to have higher mAP scores although this same model would perform poorly in the field where there are many different species and terrain conditions.

Chalmers employed a Faster R-CNN architecture, which ran inference on video streamed from their drone to a laptop. Their model used transfer learning pre-trained on the COCO dataset, which helped when they had a smaller dataset, whereas we trained our model from scratch. In terms of speed and resources, their model was able to operate at two frames per second (fps) on an Nvidia Quadro RTX 8000 GPU. This hardware costs around $10,000. Although not a direct comparison, due to the difference in target species and dataset composition, in contrast our YOLOv5l6 can potentially achieve comparable results while running on cheaper hardware (Jetson NX has an MSRP of $400) and doing inference faster, at 30 fps.

Eikelboom et al. (2019) trained their model to identify zebra, giraffe and elephant. They achieved an overall mAP of 0.77 with AP 0.81 on elephant, their highest performing class. Similar to Chalmers et al. (2019), their model had an easier time detecting objects because they were detecting larger objects, which did not suffer acutely from the small object problem. They also had more images per class (1,319 for elephant, 1,109 for giraffe and 1,877 for zebra compared to our images per class listed in Table 3). They also used a slightly more lenient IoU threshold of 0.3. Their lower IoU threshold meant that the predicted bounding boxes only needed to overlap 30% of the ground truth boxes to be considered true positives, compared to 50%, our default IoU threshold. Our most competitive AP to their 0.81 AP for the elephant class was our rhino class of 0.81 AP. Their model was a RetinaNet (Lin et al., 2017b) detector with longer inference time of 1.5 s per image (0.67 fps) whereas our Jetson can infer 30 images per second.

Our model scored highly in terms of performance and resource costs. We were able to achieve faster inference time at 30 fps (15 times faster than the fastest of the two models being compared) using a less powerful device. There are several factors affecting this speed increase. Firstly, the YOLO model is lightweight, this alone provides a considerably faster inference time than either of the models used in the comparable studies. Secondly, we used half-precision weights (16 bit floating point numbers) for our model, which speeds up inference time as discussed in the Object Detection Model section. Lastly, we must acknowledge the rapid improvement of technology in the computer vision space. Since these papers were published, YOLOv4 and YOLOv5 have been made available, both of which perform significantly better than their predecessors. The hardware has also advanced. The GPU that device that we are using did not become available until March of 2020, after these papers were published. By combining these advances in technology, our study has improved both cost and speed of wildlife object detection systems.

### Choice of performance metrics

We chose the evaluation metric AP for our model since it is most commonly used in object detection models. Other metrics such as F2 are used by some researchers to assess performance of detection rates depending on what they prioritize. F2 favors higher recall and forgives lower precision. We chose to report the most used IoU threshold of 0.50 while other researchers have used reporting thresholds as low as 0. Counting the accurate detections from an IoU of 0 allows these researchers to compare between their CNN model and manual human detection against their ground truth labels with a priority of recall over precision (Duporge et al., 2021). We avoided a very low IoU threshold for reporting. There are potential degenerate cases where a model does not detect the visual features of the target object but simply returns bounding boxes based on features near where such objects tend to be found.

### Dataset composition and training strategy

We performed class selection deliberately based on the idea that the model would be primarily used for rhino detection. Although rhino detection provided a focus of the project, we included multiple classes of megafauna because doing so offered better rhino detection. We discuss this in the Model Development section. We also used background images for up to 10% of the training dataset to reduce false positives.

We addressed a relative paucity of data by using data augmentation techniques but were also constrained by the exponential increase in the amount of compute resources required for GAN and model training. Generating GAN images requires more computing resources in addition to computing resources needed for model training. We also noted that generating GAN images of small objects often did not result in quality outputs, as the GAN generators had a relatively small feature space to learn from. Instead, we observed a greater improvement with tiling.

Although not used in this study, the model performance could be further boosted by implementing tiling during the inference stage (Ünel, Özkalayci & Çiğla, 2019). Kellenberger, Marcos & Tuia (2018) used curriculum learning, where models are exposed to more difficult training examples only after learning easier ones. In their study this dramatically reduced the number of false positive detections. Another technique that we did not explore but has been used by other researchers in the field is transfer learning (Chalmers et al., 2019; Kellenberger, Marcos & Tuia, 2018). This technique helps compensate for a lack of training data by starting out with a model feature extractor (backbone) that has been trained on a larger dataset such as COCO instead of starting out from scratch.

### Accommodating internet connectivity limitations for real-time inference

One considerable barrier to full implementation was the lack of reliable cellular connectivity in the area that the drone would be deployed. To accommodate our internet connectivity limitations, we opted for a machine learning model that runs locally and sends lightweight notifications to a message queue rather than streaming our video to the cloud for inference. The Jetson NX edge device performs inference on the drone during flight and only sends the detected video frame to the cloud. This avoids the high network cost from sending the entire live stream over a network and makes inference possible when network connectivity is unreliable.

In this study, we were able to run the inference model on the drone as described in our Results section, but we did not actually mount it on a drone due to budgetary constraints. The full implementation would mount the Jetson on a drone with a dedicated power source and cellular modem to communicate out results; these are both relatively inexpensive pieces of commodity hardware. Zhang et al. (2019) have already demonstrated the feasibility of attaching the Jetson to a drone for use in real-time inference.

A potential issue that we noticed in our test implementation is that the number of notifications can become overwhelming when an object is spotted. Running at 30 fps inference speed, we had enough data coming in that the web app we developed to show animal locations became overloaded. This volume of notifications would also overwhelm a human operator. To combat this, we recommend reducing the number of frames that are used to generate notifications. This rate could be adjusted based on the flying speed and height of the drone and the camera’s field of focus such that each square meter of ground appears in multiple frames. This would give the model multiple attempts to capture a given object of interest without sending out a gratuitous number of notifications for every object that passes through the drone’s field of view. Mulero-Pázmány et al. (2014) present a series of equations to help determine the appropriate frame rate.

### Avoiding drone disturbance to wildlife

There is ongoing debate about the appropriate altitude AGL to fly drones for conservation (Mulero-Pázmány et al., 2014; Mulero-Pázmány et al., 2017; Chalmers et al., 2019). Any disturbance to animals will depend on several factors including species sensitivity, the noise generated by the specific aircraft, wind and air pressure variables, direction of approach, *etc*. Flying lower will result in higher quality images and greater detection rates with the drawback that it potentially disturbs the animals and has less ground coverage per flight. Flying higher will cover more ground and is less disruptive to the animals but the footage captured results in the animals being small objects, making accurate detection more challenging. Based on our experience at Kuzikus, we propose that a flight altitude of 30 to 40 m AGL is a suitable compromise between these two extremes for the purpose of rhino detection. In the future, we may be able to further reduce disturbance to wildlife by carrying out remote sensing with higher-flying drones equipped with higher-resolution cameras.

We hypothesize that specialized models for different flight types (based on altitude AGL) would allow the respective models to generalize better to their captured imagery, and lead to better model performance. Even though we had distinct data sources from fixed wing (high altitude) and quadcopter (lower altitude) drones, we did not test this hypothesis due to the limited quantity of images within our dataset for each altitude. Training individual models specifically for high and low altitudes would likely help the model learn the task more efficiently and perform better than a model that combines a range of altitudes with a limited dataset.

## Conclusions

Overall, we have demonstrated the successful development of a remote sensing technique applicable for monitoring wildlife in low resource settings, where internet access is unreliable and the ability to collect large datasets may be limited. Our system is based on a fast and lightweight object detection model, which uses a suite of data augmentations to compensate for a relatively small training dataset. The model performs comparably to other published studies in terms of accuracy while having inference times that are an order of magnitude faster and running on cheaper hardware. We have also demonstrated a proof-of-concept edge implementation of a pipeline with a web app to guide potential real-world deployment. The combination of our model and implementation is ideal for low resource settings because a small edge device would be able to contain the lightweight YOLO model that can rapidly ingest and perform inference on captured imagery as the drone flies over large areas. Wildlife managers can be notified of animal locations without the need to have a human reviewing all the footage or waiting until the flight is finished to upload video to an object detector.

For researchers and wildlife managers interested in developing similar real-time wildlife detection systems on drones, we have shown that modern hardware and open-source software are capable of the task in an on-board edge device. With small adjustments, the same basic system could be adapted to other contexts. For example, Eikelboom et al. (2019) discussed methods to get accurate population estimates from object detection models run on drone footage. Further work could also focus on how to integrate this type of pipeline into a whole system. For example, determining the optimal number of drones to fly, the schedule to fly them, and the optimal flight path to maximize scarce resources on a wildlife preserve is a non-trivial problem. It depends on local conditions, the animal of concern, and whether the goal is anti-poaching, animal census, or some other tasks. The study by Park et al. (2015) covered some of these issues at a high level in the poaching context. Even when the context is determined, many low-level considerations around personnel training and operations need to be resolved before wildlife managers can implement such a system in practice.

Drones have already demonstrated their huge potential for monitoring a range of wildlife, but, however seductive, new technology for wildlife monitoring must always be assessed in terms of practical application on the ground. For example, consideration must be given to unintended use. An ‘eye in the sky’ in the hands of poachers could put rhino and other endangered species at increased risk. Effective wildlife security depends on a strongly motivated local community. Well-equipped and trained park rangers have always been the foundation of good rhino protection, and this remains true even in the presence of the most advanced remote sensing techniques.

## Supplemental Information

10.7717/peerj.13779/supp-1 Supplemental Information 1 Training image script. The script used to break the training images into tiles. Intended to be opened and run in a Jupyter notebooks environment. Relevant instructions for use are in the notebook itself.

10.7717/peerj.13779/supp-2 Supplemental Information 2 The requirements for image tiling. A text file required by TileNoteBook.ipynb that contains a list of the python libraries that are required for that script to work.

10.7717/peerj.13779/supp-3 Supplemental Information 3 Appendix: Definition of terms. Adapted from Lürig et al. (2021).

## Additional Information and Declarations

### Field Study Permissions

The following information was supplied relating to field study approvals (*i.e*., approving body and any reference numbers): Permission was granted by the National Commission on Research, Science and Technology of Namibia for the work undertaken at Kuzikus Wildlife.

### Data Availability

The following information was supplied regarding data availability: The data is available at Zenodo: Alice Hua, Kevin Martin, Zoe C Jewell, Eugene Shen, Nicole Chen, Catherine Mou, Maximilian Sterk, Berend Reinhard, Friedrich Reinhard, Stephen Lee, & Sky Alibhai. (2021). Megafauna Identification Training Images. https://doi.org/10.5281/zenodo.6386809.
