# Supplementary material for: Protecting endangered megafauna through AI analysis of drone images in a low-connectivity setting: a case study from Namibia
Source: PeerJ. 2022 Aug 3;10:e13779. doi: 10.7717/peerj.13779 (PMC9356584; doi:10.7717/peerj.13779)
Supplement: Supplemental Information 3 — Adapted from Lürig et al. (2021). [file peerj-10-13779-s003.pdf]

1. Definition of terms. adapted from Lurig et al., 2021.

Artificial Intelligence (AI): The capability of a machine or computer device to emulate human intelligence (cognitive process), refine models from experience, adapt to the latest information and operate humans-like-activities. It includes the following:

Computer Vision (CV): The process of automated extraction and processing of information from digital images. This is often implemented using machine learning techniques.

Machine Learning (ML): A subfield of AI, is the study and implementation of computer algorithms that improve automatically through experience.

Neural networks: A subset of Machine Learning algorithms made up of interconnected units (like neurons) that process information by responding to external inputs and relaying information between each unit. The process requires multiple passes at the data to find connections and derive meaning from undefined data.

Deep Learning: uses huge neural networks with many layers of processing units, taking advantage of advances in computing power and improved training techniques to learn complex patterns in large amounts of data. Common applications include image and speech recognition

Label smoothing: A technique developed by Szegedy et al. (2016) whereby a certain amount of uncertainty is added to classification labels during training. Instead of predictions being perfectly correct or incorrect, they are only mostly correct if predicted correctly and only mostly incorrect if predicted wrong. This regularization technique helps prevent the model from overfitting and lets it generalize better to unseen data.

Albumentations: A library written in the python language to help automate online image augmentation (Buslaev et al., 2020).

Offline Data Augmentation: Creating images based on existing images in the data set then storing them on disk for use in training the model.

Tiling: Breaking a large image into smaller m x n pixel images. These smaller images are then used for training instead of the large one. This allows small objects to be seen more clearly and therefore their features are learned more robustly.

Online Data Augmentation: Applying image transformation at training time without saving to disk and using the transformed images as additional training images.

Random Flipping: A mirror transformation of an image about its x-axis, y-axis, or both x and y. This allows a model to learn relevant features in a way that isn’t dependent on orientation.

Blurring: A transformation that applies a gaussian blur of varying intensity to an image. This transformation makes a model more robust and able to detect out of focus or movement blurred objects

Mix-up: A transformation that superimposes different images on top of each other. This tends to make the model stronger because it can detect fainter images and it removes some of the dependency on the specific background for the objects of interest (Zhang et al. 2017).

Mosaic: A transformation in which different images are put next to each other (e.g. in an m x n grid) before ingestion by the model. This introduces different contexts into the same image and tends to reduce context dependence for detected objects. It has been used since YOLOv4 (Bochkovskiy et al., 2020).

Image Saturation: A transformation in which the saturation channel of an image is randomly lowered. The saturation of an HSV image can be thought of as the "amount" of color. So, reducing the saturation makes the image closer to black and white. This helps reduce context dependence for the image. This is important because the color of the ground in our study varies from a rust red soil in the dry season to a lush green in the wet season.

GAN: Generative Adversarial Network is an architecture to train two neural networks (generator and discriminator) against each other in order to achieve a desirable state. In the context of image processing, the desirable state is when the discriminator can no longer classify the difference between what the generator makes, a fake image, and the original image. The discriminator is a classifier which can be trained in a supervised manner. The generator takes random noises and generates images. This type of architecture is adversarial in nature because the descriminator's goal is to lower its error rate while the generator's goal is to increase the descriminator's error rate. This relationship forces the generator to improve its generated images to the point of indistinguishable for the discriminator. The generator is then effectively a neural network that can use random noise to generate images as close to the training dataset as possible. Thus, GAN can be used for image augmentation and increase the training dataset size.

CNN: A Convolutional Neural Network is a type of state of the art deep learning model often used in computer vision. CNNs work on the principle of convolutions, where features (pixels in the computer vision) are iteratively interacted with their neighbors in the layers of the model and complex local features such as shapes, textures, and edges can be learned. Goodfellow, Bengio & Courville (2016) offer a detailed discussion of this model type.

Object Detection Algorithm: A computer vision algorithm that determines the location of objects within an image and also classifies these objects into one of a number of classes. The specific classes are determined by how the algorithm was trained.

YOLO: You Only Look Once is an object detection algorithm originally developed by Redmon et al. in 2016. It has since had four major version updates (YOLOv2, YOLOv3, YOLOv4 and YOLOv5). Three of these were published with accompanying papers (YOLOv2, YOLOv3 and YOLOv4). Shortly after YOLOv4 was published a fork of the model was created using the popular PyTorch library and named YOLOv5. The YOLOv5 library was used in our research. There is no research paper for YOLOv5 yet, but the code is publically available (Jocher et al. 2020) and the model is widely used. The YOLO family of models are notable for their speed. Unlike many of the previous state of the art models such as Faster R-CNN, YOLO models are one-stage detectors which determine object classes and bounding boxes at the same time. Although they are faster and

lighter than similar two-stage detectors, YOLO models are generally acknowledged to be less accurate than their two-stage counterparts. Our study used the YOLOv5 algorithm.

R-CNN: Region-based Convolutional Neural Networks are a family of object detection algorithms originally developed by Girshick et al. (2014) and improved on by later researchers. The most commonly used version of these is the Faster R-CNN model (Ren et al., 2017). This family of models detects objects in two-stages. First a number of candidate object boundaries are determined, then an object classification algorithm is run on the candidate boundaries to determine if there is an object of interest there and what class it is. These models generally are slower and larger than similar one-stage models, but they continue to be widely used because of their superior accuracy.

Edge device: A low-power device (i.e. sensors) that can process data close to its source (edge). In our use case, the edge device (Jetson NX) has enough computing resources to run object detection model inference at the same time the data is being collected (on the drone with this hardware attached/embedded).
